# Substrate clustering potently regulates the activity of WW-HECT domain–containing ubiquitin ligases

**DOI:** 10.1074/jbc.RA117.000934

**Published:** 2018-02-20

**Authors:** Thomas Mund, Hugh R. Pelham

**Affiliations:** From the Medical Research Council Laboratory of Molecular Biology, Cambridge Biomedical Campus, Cambridge CB2 0QH, United Kingdom

**Keywords:** ubiquitin ligase, ubiquitin, protein degradation, protein folding, protein motif

## Abstract

The Nedd4 family of HECT domain–containing E3 ligases ubiquitinate many transcription factors and signaling proteins, and their activity is tightly regulated. Normally, intramolecular interactions curb the catalytic activity of the HECT domain, but these can be broken by the binding of PY motifs, found on substrate molecules and adaptors, to the WW domains characteristic of this E3 ligase family. This raises the prospect of substrates automatically activating the ligases, frustrating the purpose of ligase regulation. Here we show that soluble protein substrates and adaptors such as α arrestins, even with multiple PY elements, cannot activate ligase activity efficiently. However, we found that polymerization or membrane tethering of these substrates dramatically increases the ligase activity both *in vivo* and *in vitro*. Aggregation of luciferase-containing substrates upon heat shock had a similar effect and could also expose cryptic PY elements in the substrates. We inferred that ligase activation critically requires a substantial array of clustered PY motifs and that the formation of such arrays on membranes or in polymeric aggregates may be an essential step in this mode of ligase regulation. We conclude that recruitment of α arrestins to membrane receptors and aggregation of unstable proteins after heat shock may be physiologically relevant mechanisms for triggering ubiquitination by Nedd4 family HECT domain–containing E3 ligases.

## Introduction

Protein ubiquitination by E1–E2–E3 multienzyme cascades controls numerous cellular processes, mainly through the induction of protein turnover via the proteasomal and lysosomal pathways (1). Substrate specificity is imparted by distinct E3 ubiquitin ligases, of which there are hundreds in mammalian cells. Although RING domain ligases are most numerous, HECT domain–containing enzymes form an important and mechanistically distinctive class (2). Of the 28 human HECT ligases, nine comprise the closely related Nedd4 family (3), characterized by an N-terminal C2 domain, two to four WW domains, and a C-terminal HECT domain, examples being Nedd4, Nedd4L, WWP1/2, Itch, and Smurf1/2 (2, 3). The WW domains bind variants of the PY motif (typically PP*X*Y), and thus proteins bearing one or more of these motifs are candidate substrates (3–5). In many cases these are membrane proteins, but Nedd4 ligases have also been implicated in the turnover of soluble proteins such as c-Jun (6) and of damaged proteins following heat shock (7). Abnormal activity of Nedd4 ligases has frequently been implicated in cancer, immune disorders, and other diseases (8–12).

To prevent constitutive ubiquitination and destruction of themselves and substrates, the activity of Nedd4 family HECT ligases is generally thought to be restrained by autoinhibition. Extensive structural studies have shown that the catalytically active HECT domain consists of two lobes that move relative to each other during the catalytic cycle; in one position, ubiquitin can be transferred from a bound E2 thioester to the active site cysteine, whereas subsequent transfer to a substrate can only occur after a large rotation of the C-terminal lobe (13–19). Autoinhibition is achieved by interactions with the HECT domain that block this conformational flexibility. For some enzymes, such as Smurf2 and Nedd4, the C2 domain is responsible (20), whereas in others, such as Itch, WWP1, and WWP2, an α-helical peptide between the WW2 and WW3 domains, in conjunction with the adjacent WW2 domain, binds between the N and C lobes and mediates the inhibition (21, 22). This regulatory mechanism is unlikely to apply to all HECT ligases, however. The HECT domain of E6AP, which lacks WW domains, is only weakly active and has been reported to require trimerization (or dimerization) for maximal activity (23), thus providing a positive regulatory mechanism. Whether some element of this alternative mechanism could also apply to the Nedd4 family ligases is currently unclear. In contrast to E6AP, their isolated HECT domains are constitutively active in self-ubiquitination. There is some kinetic evidence for co-operativity between molecules in the formation of free polyubiquitin chains (24), although this reaction may be different from the sequential addition of monomeric ubiquitin to substrates, which appears more typical for Nedd4 enzymes (25).

Activation of the Nedd4 family ligases, and guiding them to specific substrates, is commonly achieved by adaptor proteins that themselves contain multiple PY motifs, including, in animal cells, the Ndfip1 and Ndfip2 proteins (6, 26, 27) and the α arrestins (28, 29), originally identified in yeast as Bsd2, and the arrestin-like Art proteins, respectively (30, 31). Binding of these to the WW domains (particularly WW2 in the case of Itch (22)) evidently disrupts the inhibitory conformation, freeing the HECT domain to act on adjacent substrates. In some cases, a similar activation effect is apparently achieved by phosphorylation (32, 33).

Activation by PY motifs presents a paradox; many substrates contain a PY sequence, but if this were sufficient to activate the ligase, then activity would effectively be constitutive, and there would be little regulation. There must therefore be additional requirements for activation if regulation is to be effective.

We recently showed that the Wnt signaling component Dvl2 is both an activator and substrate for the Nedd4 family member WWP2 (34). Dvl2 is a soluble protein containing a single PY motif sufficient for interaction with WWP2. However, activation of the ligase occurs only when Dvl2 is polymerized, which happens normally in response to Wnt signaling. This system has evolved to facilitate intracellular signaling, and there are multiple interactions between Dvl2 and WWP2.

Here we have investigated more widely the effects of polymerization, aggregation, and membrane tethering on the ability of PY-containing proteins to activate and become substrates for Nedd4 family HECT ubiquitin ligases. We show that soluble proteins bearing one or a few PY motifs are very poor substrates. However, their polymerization or membrane tethering can dramatically enhance ligase activation. This indicates that a high local density of PY motifs is an essential component of the activation process. Aggregation of PY-containing proteins following heat shock can also promote ubiquitination, and this may help to explain how Nedd4 family ligases contribute to the degradation of damaged proteins.

## Results

### Activation by membrane-tethered Dvl2

Many substrates for Nedd4 family ligases are integral or peripheral membrane proteins, which are restricted to two dimensions and may also be locally clustered. In contrast Dvl2 is a soluble protein, and our previous study showed that it becomes a substrate only when assembled into large particles containing many molecules. A possible interpretation is that clustering and/or restricted mobility is the key feature required for ligase activation and that membrane anchoring and polymerization are alternative ways to achieve this. If so, the polymerization requirement for Dvl2 might be circumvented by attaching it to the membrane.

**Figure 1.**
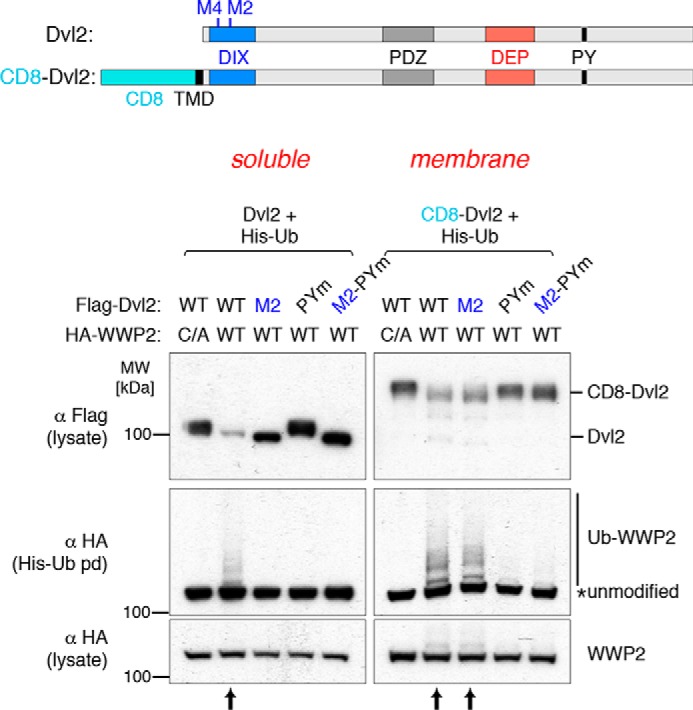
**Ligase activation is polymerization-independent in membrane-tethered dishevelled.** Shown are Western blots of His pulldowns from HEK293T cells co-transfected with His-Ub, FLAG-Dvl2 (WT, M2, PP*X*Y mutant, or M2-PP*X*Y mutant) and HA-tagged WT or catalytically dead (*C/A*) WWP2, as indicated above the panels. The *left panel* shows the experiment using soluble Dvl2 compared with membrane-targeted CD8-Dvl2 (*right panel*). WWP2 autoubiquitination is induced by WT but not PP*X*Y mutant or polymerization-defective (M2) Dvl2; membrane-targeted Dvl2 induces WWP2 autoubiquitination and degradation even in the absence of polymerization (CD8-M2-Dvl2) (*arrows*). The blots are representative of three independent experiments. *MW*, molecular weight.

We co-expressed Dvl2 and WWP2 with His-Ub^2^ and monitored ubiquitination by pulldown of the His-tagged species and immunoblotting. This resulted in activation of WWP2, revealed by its autoubiquitination, and also degradation of Dvl2 (Fig. 1). Both are abolished by the M2 mutation in the Dvl2 DIX domain, which prevents polymerization (35), as well as by mutations that inactivate the ligase activity of WWP2 or the PY motif of Dvl2 (Fig. 1). We then fused Dvl2 to the extracellular and transmembrane domains of the plasma membrane protein CD8 and found that this resulted in equally efficient activation of WWP2. In this case, the M2 mutation had no effect, although the PY motif was still essential (Fig. 1, *right panels*). This shows that membrane tethering is indeed an alternative to polymerization in providing PY-dependent ligase activation by Dvl2.

### Polymerization substitutes for membrane tethering of Ndfip

**Figure 2.**
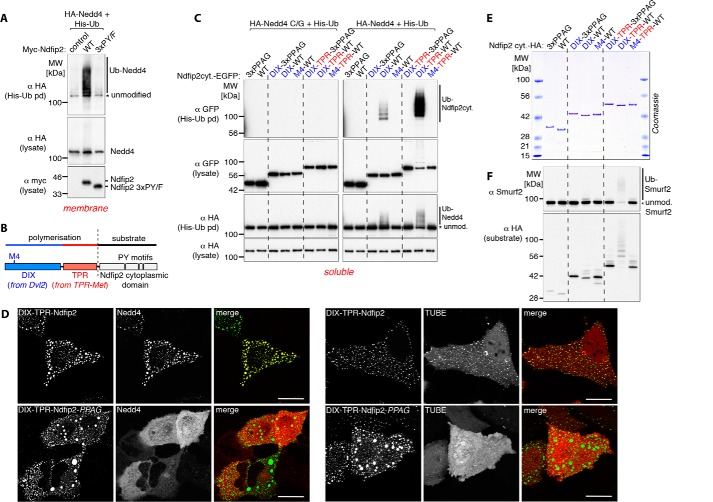
**Soluble aggregated proteins activate Nedd4 ligases.** *A*, Western blots of His pulldowns from HEK293T cells co-transfected with His-Ub, myc-Ndfip2 (WT or PP*X*Y mutant), and HA-tagged Nedd4, showing efficient ligase activation with the full-length endosomal membrane protein Ndfip2. *MW*, molecular weight. *B*, schematic of the polymerization domains (DIX and TPR) fused to a soluble substrate (the cytoplasmic domain of Ndfip2 containing three PY motifs) used in this study. *C*, Western blots of His pulldowns from HEK293T cells co-transfected with His-Ub, HA-tagged catalytically dead (*C/G*) or WT (*right panels*) Nedd4 and the GFP-tagged cytoplasmic domain of Ndfip2 alone or fused to the weakly polymerizing DIX or the highly efficient polymerizing DIX-TPR, as indicated above the panels. The soluble cytoplasmic domain of Ndfip2 efficiently activates Nedd4 autoubiquitination only with intact PY motifs and when fused to intact DIX-TPR. *D*, live-cell images showing single confocal sections of representative HeLa cells co-transfected with DIX-TPR-Ndfip2-EGFP (intact PY motifs or PPAG mutants) and mCherry-Nedd4 (*top*) or mCherry-TR-TUBE (*bottom*), demonstrating efficient recruitment of Nedd4 and the ubiquitin sensor TUBE into the punctate aggregates in the presence of intact PY motifs. *E*, Coomassie-stained gel showing purified HA-tagged recombinant proteins used in *F*, indicated above the panel. *F*, *in vitro* ubiquitination assays using Smurf2 with HA-tagged purified recombinant proteins (*E*) as indicated above the panels, showing efficient Smurf2 autoubiquitination only when incubated with polymerization-competent DIX-TPR and of intact PY motifs. All blots are representative of two or more similar independent experiments. *Scale bars* = 10 μm.

If polymerization and membrane anchoring are equivalent, then a prediction is that PY motifs from a membrane protein activator would not function in soluble form but would do so when polymerized. We and others have shown previously that Ndfip1 and Ndfip2 are efficient natural activators of Nedd4 ligases (6, 36) (Fig. 2*A*). They are membrane proteins with a cytoplasmic N-terminal sequence containing three PY motifs (Fig. 2*B*), which are required for activation. Fusion of this cytoplasmic domain to soluble GFP did not result in any detectable activation of co-expressed Nedd4 or any ubiquitination of the GFP fusion (Fig. 2*C*, *right panel*, *WT*). Addition of the Dvl2 DIX domain to the construct did result in some activation, shown by ubiquitination of both the ligase and the GFP-Ndfip construct. This was abolished by the M4 mutation, which, like the M2 mutation, prevents DIX polymerization (Fig. 2*C*). Although the DIX domain is capable of self-association, it alone polymerizes inefficiently; efficient formation of large aggregates requires an additional dimerization domain, a function that can be provided by the TPR (translocated promoter region) domain from TPR-Met (Fig. 2, *B* and *C*, and Refs. 35, 37). Addition of this domain resulted in very efficient activation of ubiquitination, which again was abolished by the M4 or PY mutations (Fig. 2*C*, *right panel*). Expression of an active site C/G mutant of Nedd4 did not result in ubiquitination, indicating that, under these conditions, modification was largely due to the expressed Nedd4 (Fig. 2*C*, *left panel*). Thus, polymerization can restore the ligase activation function of Ndfip2, which is otherwise dependent on membrane anchoring.

Fluorescence microscopy of cells expressing these constructs clearly showed the formation of punctate cytoplasmic structures to which Nedd4 was recruited and on which ubiquitin was detected (Fig. 2*D*). These are evidently stable structures rather than loose aggregates, as photobleaching of a small region was not followed by rapid recovery of fluorescence (Fig. S1, *A* and *B*). Mutation of the Ndfip PY motifs did not affect puncta formation but abolished recruitment of Nedd4, showing that they are indeed the binding sites.

Previously, we and others showed that multiple PYs were required for Ndfips to function effectively (26, 36). These results show that this is not sufficient; polymerization, or restriction to membranes, is important even for sequences that contain multiple PY motifs. Evidently it is not just the presence of linked PY motifs but rather the provision of a dense local array of such sequences that is necessary for efficient activation of Nedd4. Furthermore, the effect appears to be general; it is not restricted to Dvl2, a complex protein that has multiple interactions with Nedd4 ligases, but can easily be reconstructed with artificial constructs.

To test more directly whether activation is solely due to direct interactions between the ligase and the PY-bearing construct, we performed experiments with purified proteins *in vitro* (Fig. 2, *E* and *F*). In such assays, high levels of the cytoplasmic domain of Ndfip2 are sufficient to activate the ligase Itch (26). However, at modest levels (three times the concentration of the ligase), this fragment did not activate Smurf2 ligase, whose activity is efficiently autorepressed (Fig. 2*F*, lane corresponding to *WT* in Fig. 2*E*). When fused to the DIX domain, it remained a poor substrate (as judged by its own ubiquitination) and inefficient activator (as judged by autoubiquitination of Smurf2). Only when the TPR dimerization domain was added did activation become efficient (Fig. 2*F*), just as for the *in vivo* experiments with Nedd4.

### Arrestin domain–containing proteins also require membrane tethering or polymerization for activity

**Figure 3.**
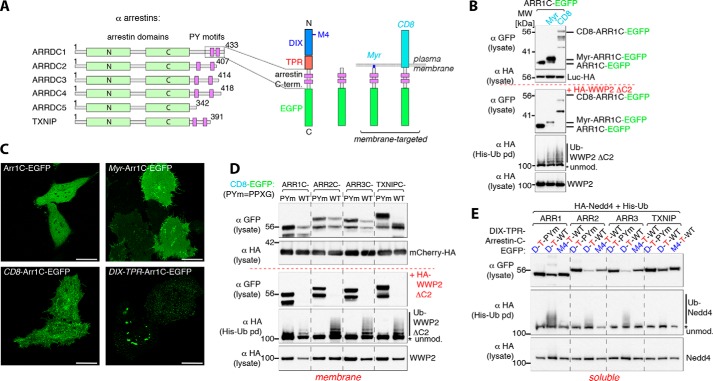
**Arrestin domain–containing proteins also require membrane tethering or polymerization for activity.** *A*, schematic of α arrestins (*left panel*) and overview of the constructs used in this study. *B*, Western blots of His pulldowns from HEK293T cells co-transfected with His-Ub, with or without (*top panel*) HA-tagged DC2-WWP2 and either GFP-tagged soluble or membrane-targeted Arr1C, as indicated above, showing efficient WWP2 activation with membrane-tethered PY motif–containing constructs. *MW*, molecular weight. *C*, live-cell single confocal sections of representative HeLa cells transfected with the constructs used in *B* or *E* (ARR1 d-T-WT). *D* and *E*, Western blots of His pulldowns from HEK293T cells co-transfected with His-Ub, with or without HA-tagged ligase, and either membrane-tethered (*D*) or soluble polymerization-competent (*E*) α arrestin constructs, as indicated, demonstrating efficient ligase activation of all tested arrestins with intact PY motifs as well as polymerization-dependent ligase activation of soluble PY motif–containing arrestin constructs. The mCherry-HA shown in the *top panel* in *D* was co-transfected to serve as a gel loading and transfection control. All blots are representative of two or more similar independent experiments. *Scale bars* = 10 μm.

The α arrestins are a group of soluble proteins typically containing a pair of PY motifs that associate with and facilitate the ubiquitination of G protein–coupled receptors (29) (Fig. 3*A*). Like the Ndfips, they act as HECT ligase adaptors, but they are not themselves integral membrane proteins. From the results above, a prediction would be that their ability to activate Nedd4 ligases is specifically triggered by their association with membranes, thus sparing them from constitutive degradation, and that this reflects clustering of the PY motifs.

In agreement with this, we found that co-expression of C-terminal PY motif–containing sequences from the arrestin ARRDC1, fused to GFP, did not activate the Nedd4 ligase WWP2 efficiently. However, anchoring the ARRDC1-GFP construct to the membrane using N-terminal fusions of either a myristylation site or the CD8 transmembrane and extracellular domains resulted in much more efficient activation and autoubiquitination (Fig. 3*B*). There was also a marked reduction in the level of the membrane-targeted constructs when WWP2 was co-expressed, consistent with their ubiquitination and degradation. In this experiment, we used a version of WWP2 that lacked its potential membrane-binding C2 domain and thus should not be preferentially associated with membranes in the absence of binding sites; removal of this domain does not in itself lead to activation of WWP2 (21). Fig. 3*C* shows that the constructs themselves were mostly on the plasma membrane, evenly distributed in the case of the myristoylated form and rather more clustered for the CD8 fusion.

More generally, Fig. 3*D* shows that C-terminal sequences from four different α arrestins could activate WWP2 in a PY-dependent manner when fused to CD8 and that this was accompanied by dramatic loss of the GFP–arrestin fusion, presumably because of their ubiquitination and degradation. Even without co-expression of WWP2, we could observe this selective degradation (Fig. 3*D*, *top panel*), suggesting interaction with endogenous Nedd4 family ligases. The PY mutations tested in Fig. 3*D* (PP*X*G) change the Tyr residue, which forms part of a putative Y*XX*Φ endocytic signal. However, the effects cannot be ascribed to changes to this signal, as alternative mutations (AA*X*Y) that do not affect it gave similar results (Fig. S1*C*).

Finally, we fused these same C-terminal arrestin sequences to the DIX-TPR construct and tested them for activation of co-expressed Nedd4. These constructs formed punctate aggregates (Fig. 3*C*) and activated the ligase, but only when they were polymerization-competent and had intact PY motifs (Fig. 3*E*). We conclude that the α arrestins do not activate Nedd4 ligases when they are soluble but can do so when they are membrane-associated and that this is likely due to clustering of PY motifs because polymerization has a similar effect.

### Heat-induced aggregates can be substrates for Nedd4 ligases

Mild heat shock results in increased ubiquitination of proteins, which is generally considered a natural response that promotes degradation of thermally denatured proteins. There is evidence that, at least in yeast, some of this is mediated by a Nedd4 family ligase (7). A possible explanation might be that heat-induced protein aggregation creates clusters of otherwise ineffective PY motifs that are sufficient to activate Nedd4 ligases and trigger ubiquitination.

**Figure 4.**
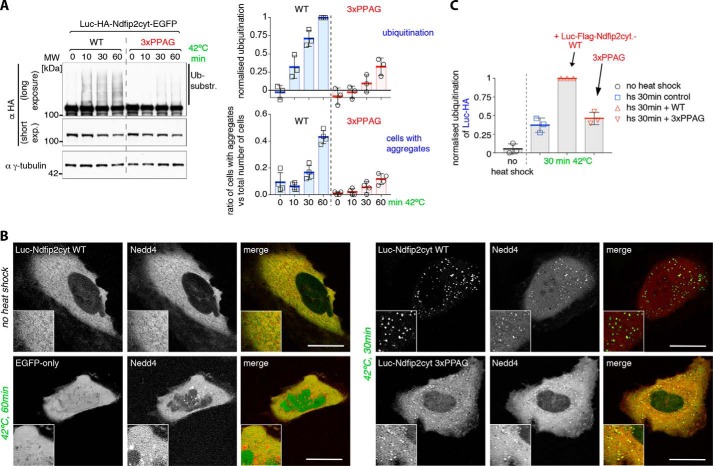
**Heat-induced aggregates can be substrates for Nedd4 ligases.** *A*, Western blots of whole-cell lysates from control or heat-shocked HEK293T cells co-transfected with His-Ub and the soluble cytoplasmic domain of Ndfip2 (WT or PY motif mutant) fused to luciferase and EGFP. Shown on the *right* are the quantification of ubiquitinated luciferase-Ndfip2 via densitometry from the blots as well as a quantification of the appearance of visible aggregates in heat-shocked cells from the same experiment, demonstrating that mutation of the Ndfip2 PY elements not only reduced ubiquitination but also reduced the appearance of aggregates. *MW*, molecular weight. *B*, live-cell single confocal sections of representative control or heat-shocked HeLa cells transfected with luciferase-Ndfip2-EGFP or EGFP-only and mCherry-Nedd4, showing recruitment of ligase into aggregates upon heat shock. *C*, quantification of Western blots of whole-cell lysates from control or heat-shocked HEK293T cells co-transfected with His-Ub, luciferase-only, and FLAG-tagged luciferase-Ndfip2 WT or PY motif mutant, as indicated, demonstrating PY motif–dependent ubiquitination of co-aggregated luciferase. All blots are representative of at least two similar independent experiments, and graphs show individual data points from different experiments with the mean and standard deviation indicated. Densitometric quantification data were obtained within a linear range of exposure, and ubiquitination levels were normalized to the highest value in each set of experiments. Scale bars = 10 μm.

To test this hypothesis, we examined the fate of constructs based on firefly luciferase, a protein that is easily unfolded by heat. We first appended the Ndfip2 cytoplasmic tail to GFP-tagged luciferase and found that we could detect PY motif–dependent ubiquitination of this construct following heat shock (Fig. 4*A*). Fluorescence microscopy revealed that the protein concentrated into visible dots following heat shock (Fig. 4*B*), which represent stable aggregates, as they did not recover after photobleaching (Fig. S1, *A* and *B*). Furthermore, when Nedd4 was co-expressed, it was recruited to these dots (Fig. 4*B*).

The ubiquitination in Fig. 4*A* is from endogenous ubiquitin ligases, presumably of the Nedd4 family. As further evidence for this, we co-expressed a dominant negative form of WWP2 with the active-site cysteine mutated. Fig. S1*D* shows that this reduced the endogenous ubiquitination of the luciferase construct relative to a control in which the WW domains were also mutated to prevent substrate interaction. This is a further indication that the heat-induced modification of these constructs is mediated by Nedd4 ligases through WW–PY interactions rather than by other types of ubiquitin ligases.

Unexpectedly, mutation of the Ndfip2 PY elements not only reduced ubiquitination but also reduced the appearance of aggregates; they were smaller, in a smaller fraction of the cells, and tended to appear later (Fig. 4, *A* and *B*). It seems that ubiquitination promotes aggregate formation. Surprisingly, the aggregates that did form still recruited Nedd4 (Fig. 4*B*), and, indeed, ubiquitination was not completely abolished. There are several possible explanations for this. The luciferase could form co-aggregates with other proteins containing PY motifs, perhaps including chaperones, as reported in yeast (7); there could be weak PY motifs in our constructs that are only accessible after protein unfolding; or Nedd4 may have an inherent tendency to adhere to exposed hydrophobic surfaces in aggregates, distinct from the specific recognition of PY motifs.

### Promotion of ubiquitination by co-expressed activators

To see whether ubiquitination mediated by co-aggregated activators could be detected, we expressed HA-tagged luciferase together with a smaller amount of FLAG-tagged luciferase bearing the Ndfip2 cytoplasmic domain. Following heat shock, ubiquitination of the HA-luciferase could be detected, and this was noticeably enhanced when the PY-containing construct was co-expressed, relative to a PY-mutated control (Fig. 4*C*). We conclude that the PY-tagged luciferase can activate Nedd4 ligases and that these can then act on other nearby substrates; in this case, co-aggregated luciferase.

### Heat shock may expose PY motifs that are otherwise inaccessible

A significant number of proteins contain PY-like sequences, many of which are presumably within folded structures and inaccessible under normal conditions. However, a combination of unfolding and aggregation could allow them to recruit ligases following heat shock.

**Figure 5.**
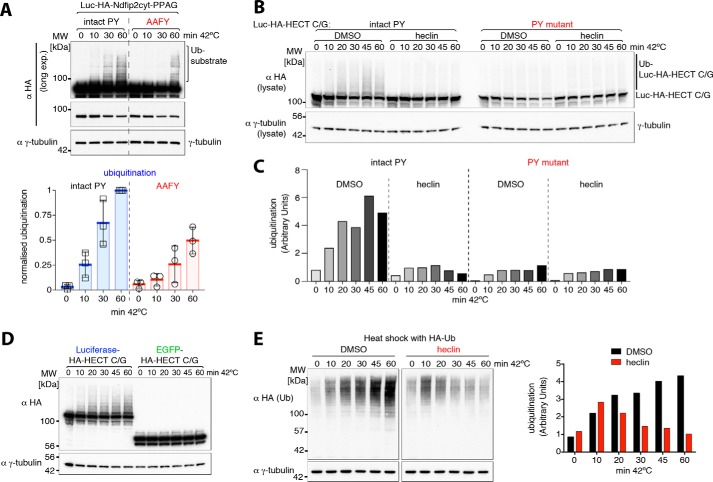
**Role of heat shock–exposed “inaccessible” PY motifs in ubiquitination.** *A*, Western blots of whole-cell lysates from control or heat-shocked HEK293T cells co-transfected with His-Ub and luciferase-Ndfip2 (3xPPAG)-EGFP or a mutant luciferase version (*AAFY*). Quantification of ubiquitinated luciferase constructs via densitometry is shown on the *right*, showing reduced and delayed ubiquitination of the AAFY mutant upon heat shock. *MW*, molecular weight. *B*, Western blots of whole cell lysates from control or heat-shocked HEK293T cells co-transfected with His-Ub and luciferase-fused inactive HECT domain with an intact or mutant (normally inaccessible) PY motif. Heat shock experiments were carried out either with 1% DMSO control or 100 μm heclin as indicated. *C*, quantification of substrate ubiquitination via densitometry shown in *B. D*, Western blots of whole-cell lysates from control or heat-shocked HEK293T cells co-transfected with His-Ub and either luciferase or EGFP-fused inactive HECT domain, showing luciferase-dependent ubiquitination upon heat shock. *E*, HEK293T cells expressing hemagglutinin (HA)-Ub were treated with DMSO control or heclin and harvested after incubation at 42 °C for the times indicated. A densitometric quantification of ubiquitination is shown on the *right*. All blots are representative of two or three similar independent experiments, and the graph in *A* shows individual data points from different experiments, with the mean and standard deviation indicated. Densitometric quantification data were obtained within a linear range of exposure, and ubiquitination levels were normalized to the highest value in each set of experiments.

Luciferase itself contains the sequence APFY, potentially a weak PY motif, and so we tested the effects of mutating this to AAFY. This did both reduce and delay ubiquitination following heat shock but did not eliminate it completely (Fig. 5*A*). Thus, weak non-canonical PY motifs may contribute to ligase activation.

One well-characterized example of a cryptic PY motif is the conserved inaccessible LP*X*Y motif in the HECT domain, which cannot be bound by a WW domain unless the protein is heated (36, 38). To test whether this could contribute to ubiquitination, we mutated the active-site cysteine of the Smurf2 HECT domain, thus making it a substrate rather than an enzyme, and fused it to luciferase to promote aggregation. Following heat shock, it indeed became ubiquitinated, and mutation of the LP*X*Y motif prevented this (Fig. 5, *B* and *C*). This ubiquitination was also blocked by heclin, a compound that is a general inhibitor of HECT ligases (39) (Fig. 5, *B* and *C*). Ubiquitination was not observed when the inactive Smurf2 HECT domain was fused to GFP (Fig. 5*D*), suggesting that it is not particularly aggregation-prone but must be associated with a protein that is. This example demonstrates that unmasking of cryptic PY elements, coupled with aggregation, is a plausible mechanism to account for at least some of the increased ubiquitination observed after heat shock.

### HECT ligases contribute significantly to heat-induced ubiquitination

If HECT ligases contribute significantly to ubiquitination following heat shock, then inhibiting them should have a detectable effect. Fig. 5*E* shows an experiment in which general ubiquitination of cellular proteins (detected by expressing HA-tagged ubiquitin) was monitored during a heat shock. At early times (10 min), increased ubiquitination could already be detected, and it was not inhibited by heclin; indeed, it was slightly more prominent when heclin was present. This is presumably mediated by other types of ubiquitin ligases, such as RING domain ones. At later times, however, there was a substantial further increase in ubiquitination that was abolished by heclin. Heclin inhibits essentially all HECT domain ligases, not just the Nedd4 family, so we can only draw the broad conclusion that HECT domains are involved (39). Nevertheless, the timing and scale of the effect are at least consistent with the hypothesis that Nedd4 ligases contribute to the later stages of the response, perhaps as small aggregates of denatured proteins are formed and ligases are recruited to them.

## Discussion

Our results, together with previous work, allow a number of conclusions to be drawn about how Nedd4 family ligases interact with their substrates. First, it is clear that the presence of one PY element, or even two or three, on a soluble protein is not sufficient for it to become a substrate for ubiquitination, even though, as we have shown previously for Dvl2, it may be able to bind to the ligase through a WW domain. Thus, for example, the α arrestins are unlikely to be ubiquitinated and degraded while free in solution. This is because the ligase first needs to be activated. This can also be mediated by PY motifs, but the requirements are more complex. Specifically, it appears that a dense array of PY elements is necessary, which can be provided either by the formation of polymers or aggregates or by them being arrayed on a two-dimensional membrane. This requirement provides a natural control mechanism. During Wnt signaling, polymerization of Dvl2 triggers ligase activation, and we suggest that recruitment of α arrestins from solution to activated G protein–coupled receptors may have a similar regulatory effect.

The activation mechanism likely involves the disruption of inhibitory interactions with the HECT domain, which needs to be flexible to function (16). For some enzymes, this involves the C2 domain (20), and for others, peptide sequences near the WW domains are involved (21, 22), but it seems that in all cases engagement of multiple WW domains with PY motifs is sufficient to maintain an open and active conformation. We initially envisaged that a 1:1 complex between the enzyme and an activator with multiple PYs would be sufficient, but it is clear that a larger polymeric array of such elements is required. Why is this? One clue is that interaction of Nedd4 with the endosomal membrane form of Ndfip2 is transient, as shown by recovery after photobleaching (26). When released, ligase may reform its inhibited conformation or, alternatively, bind again to the PY array. We suggest that a high local concentration of binding sites results in rebinding being more rapid than inhibition, resulting in continuously active enzyme. In contrast, at lower concentrations of activator, reinhibition would be the kinetically determined predominant outcome.

An alternative possibility is that it is the adjacency of multiple enzyme molecules that promotes activation. This is reminiscent of the trimerization-dependent activation exhibited by E6AP (23) but is unlikely to be completely analogous. Isolated monomeric HECT domains of the Nedd4 family are constitutively active rather than inactive, implying negative regulation, and high concentrations of soluble PY-containing peptides efficiently activate the Nedd4-like Itch enzyme (26), conditions that are unlikely to favor oligomerization. Nevertheless, interaction with or preferential trans-ubiquitination of adjacent enzyme molecules may well contribute to enhanced activation under clustering conditions.

Regardless of the precise mechanism, the effects of clustering are striking, and they show the power of a multitude of weak binding sites. Such phenomena may be quite widespread in biology.

Our results also show that heat shock, through the unfolding and aggregation of sensitive proteins such as luciferase, can provide another physiologically relevant mechanism to create arrays of PY motifs sufficient to activate Nedd4 ligases, resulting in ubiquitination and eventual disposal of the damaged proteins. It is difficult to assess how physiologically significant this might be, but PY-like motifs are quite common; for example, 22% of yeast open reading frames contain a match to (PLAVI)P*X*Y, half of which are PP*X*Y or LP*X*Y motifs. We have demonstrated the principles both of conformational unmasking of a cryptic PY element and the trans-ubiquitination of co-aggregated proteins by activated ligase. Studies in yeast have given similar results, with examples of both conformational unmasking of PY elements and indirect recruitment of ligase via a PY-containing dnaJ chaperone (7). Together, these effects could contribute significantly to the cellular response to heat-induced damage.

An unexpected finding is that aggregation and ubiquitination appear to be mutually interdependent in that visible aggregation of luciferase-based constructs is more rapid and efficient when ubiquitination is enhanced by the provision of PY motifs. This could be due to the physical properties of long ubiquitin chains, such as a tendency to associate, but it could also be facilitated by ubiquitin-binding proteins. The predicted consequence is that the process of aggregation and ubiquitination should be autocatalytic; clustering promotes ligase activation, which promotes ubiquitination, which promotes further aggregation. Ligases such as Nedd4 contain ubiquitin binding sites on their HECT domains (40, 41), and this may also contribute to their association with, and activation by, existing polyubiquitin chains. Such an autostimulatory phenomenon may explain why, even when obvious PY motifs are removed, our luciferase constructs eventually become ubiquitinated, and at least some cells accumulate visible aggregates, and these, in turn, recruit ligase (*e.g.* Fig. 4*B*). It is also consistent with the delayed appearance of heclin-sensitive (and thus HECT-mediated) protein ubiquitination during heat shock.

Of course, there are many ubiquitin ligases in the cell, and we have shown that a substantial portion of the ubiquitination that initially follows heat shock is resistant to heclin and, thus, is presumably carried out by non-HECT ligases. How these various ubiquitination events combine to protect the cell remains to be elucidated.

## Experimental procedures

### Plasmids

The following plasmids have been described before: FLAG-Dvl2, FLAG-M2-Dvl2, FLAG-M4-Dvl2, FLAG-Dvl2-Pym, HA-tagged NEDD4 family ligases (WT and catalytically inactive mutants), His-ubiquitin, HA-ubiquitin, and NDFIP2 (26, 34, 39, 42). All polymerization constructs were cloned into pEGFP-N1 (Clontech). They contained various combinations of the DIX domain (aa 1–114 of human Dvl2), TPR (aa 74–141 of TPR-Met), and the cytoplasmic domain of mouse NDFIP2 containing the three PY motifs (aa 1–117, UniProtKB Q3V1V0). For live-cell imaging studies, Nedd4 and the trypsin-resistant tandem ubiquitin binding entity TR-TUBE (43) were N-terminally tagged with mCherry. The following PY motif–containing C-terminal arrestin fragments were cloned with or without polymerization domains into pEGFP-N1 (Clontech): Arrdc1 (aa 391–433), Arrdc2 (aa 330–407), Arrdc3 (aa 338–414), and TXNIP (aa 317–391). Two sets of PY motif mutants were used: PP*X*G and AA*X*Y. Plasma membrane-targeting constructs had either the N-terminal myristylation sequence MGCGCSSHPEDDGGSGGSGGS or contained residues 1–206 of human CD8A. All heat shock–sensitive constructs contained firefly luciferase with various combinations of the following: a triple HA or single FLAG tag, WT or PY motif mutant (3xPPAG) NDFIP2 (aa 1–117), an inactive C/G mutant Smurf2 HECT domain (aa 371–748) with an intact or mutated PY motif (IPPG), and/or a C-terminal EGFP. For bacterial expression, full-length Smurf2 was cloned into pGEX6P-2 (GE Healthcare). All polymerization constructs were cloned into a modified pET28a vector (Novagen) with a C-terminal triple HA tag, a PreScission cleavage site, glutathione *S*-transferase (GST), and a His tag. Truncations and point mutations were generated by standard procedures and verified by sequencing.

### Cell transfection and cell-based assays

HEK293T cells (acquired from the ATCC) were cultured and transfected essentially as described previously (26, 34, 39). Also, a 293T-derived Dvl1–3 knockout cell line has been used to verify the non-detectable effects of endogenous dishevelled on degradation and ubiquitination of overexpressed polymerization constructs containing the DIX domain (44). Transient transfections of 293T cells were performed using polyethyleneimine (PEI MAX, linear, molecular weight 40,000, Polysciences, Warrington, PA). Typically, for 2 μg of plasmid DNA, 5 μl of a 1 mg/ml solution of PEI was used. Cells were harvested about 24 h after transfection. His-ubiquitin pulldowns were done essentially as described previously (34, 39). Briefly, His-ubiquitin pulldowns were done under denaturing conditions. Typically, 1 × 10^6^ cells were harvested in 0.8 ml of 8 m urea, 100 mm Tris (pH 7.4), 2 mm *N*-ethylmaleimide, and 10 mm iodoacetamide, sonicated, and incubated with 20 μl of His-Tag Dynabeads (Life Technologies) at room temperature for several hours. The beads were washed three times in 8 m urea and eluted in sample buffer. Immunoblotting was done using standard procedures, and proteins were detected using antibodies from Sigma-Aldrich (HA, FLAG M2, γ-tubulin, and myc), Roche (GFP), or Abcam (Smurf2 and ab53316). Heat shock experiments were carried out in a 42 °C water bath, and cells were harvested by adding 0.8 ml of 8 m urea, 100 mm Tris (pH 7.4), 2 mm *N*-ethylmaleimide, and 10 mm iodoacetamide. For live-cell imaging, HeLa cells were seeded into chambered coverglass (Lab-Tek) and transfected with either polyethyleneimine (PEI MAX, linear, molecular weight 40,000) or FuGENE HD transfection reagent (Promega). Confocal images were captured on a Zeiss 510 LSM using standard filter sets.

### Recombinant protein expression and purification

All recombinant proteins were expressed as GST fusions. Plasmids were transformed into BL21 (DE3) CodonPlus-competent *Escherichia coli* (Stratagene), and expression was induced with 0.5 mm isopropyl 1-thio-β-d-galactopyranoside at 18 °C overnight. Cell pellets were resuspended in PBS containing 0.5 mm tris(2-carboxyethyl)phosphine and 5% glycerol and lysed by sonication. GST fusion proteins were affinity-purified from soluble bacterial lysates by using glutathione–Sepharose (GE Healthcare) according to standard manufacturer's protocols and cleaved off GST while bound to the beads by 2-h incubation at 30 °C with PreScission protease (GE Healthcare). Soluble recombinant proteins were either concentrated with Amicon Ultra centrifugation filter devices (Millipore) and stored as 5–10 mg/ml stocks at −80 °C until required (Smurf2), or the concentration was adjusted to about 0.5–1 mg/ml (polymerization-competent proteins) and stored at −80 °C.

### In vitro ubiquitination assays

*In vitro* ubiquitination experiments were set up essentially as described before (39). Assays (30 μl) typically contained 150 ng of human E1 (BostonBiochem, Cambridge, MA), 150 ng of the human E2 (UbcH5c, BostonBiochem), 10 μg of ubiquitin (BostonBiochem), 100 ng of Smurf2, and 200 ng of substrates with varying degrees of polymerization competence. Assays were incubated at 30 °C for 60 min and stopped by adding sample buffer.

## Author contributions

T. M. and H. R. P. conceptualization; T. M. investigation; T. M. methodology; T. M. and H. R. P. writing-review and editing; H. R. P. supervision; H. R. P. writing-original draft.

## Supplementary Material

Supporting Information

## Footnotes

2 The abbreviations used are:

Ub: ubiquitin
HA: hemagglutinin
aa: amino acids
EGFP: enhanced GFP
GST: glutathione *S*-transferase
PEI: polyethyleneimine.
