# Supplementary material for: Substrate clustering potently regulates the activity of WW-HECT domain–containing ubiquitin ligases
Source: J Biol Chem. 2018 Feb 20;293(14):5200–9. doi: 10.1074/jbc.RA117.000934 (PMC5892558; doi:10.1074/jbc.RA117.000934)
Supplement: Supporting Information [file supp_293_14_5200__index.html]

Substrate clustering potently regulates activity of WW-HECT domain-containing ubiquitin ligases — Activation of ubiquitin ligases by substrate polymerisation — Substrate clustering potently regulates the activity of WW-HECT domain–containing ubiquitin ligases — Activation of ubiquitin ligases by substrate polymerization — Supporting Information 

# Substrate clustering potently regulates the activity of WW-HECT domain–containing ubiquitin ligases

## Supporting Information

- Figure S1 - Figure with legend
