# Supplementary material for: Substrate clustering potently regulates the activity of WW-HECT domain–containing ubiquitin ligases
Source: J Biol Chem. 2018 Feb 20;293(14):5200–9. doi: 10.1074/jbc.RA117.000934 (PMC5892558; doi:10.1074/jbc.RA117.000934)
Supplement: Supporting Information [file supp_RA117.000934_133876_1_supp_76596_p4gdvt.pdf]

SUPPORTING INFORMATION

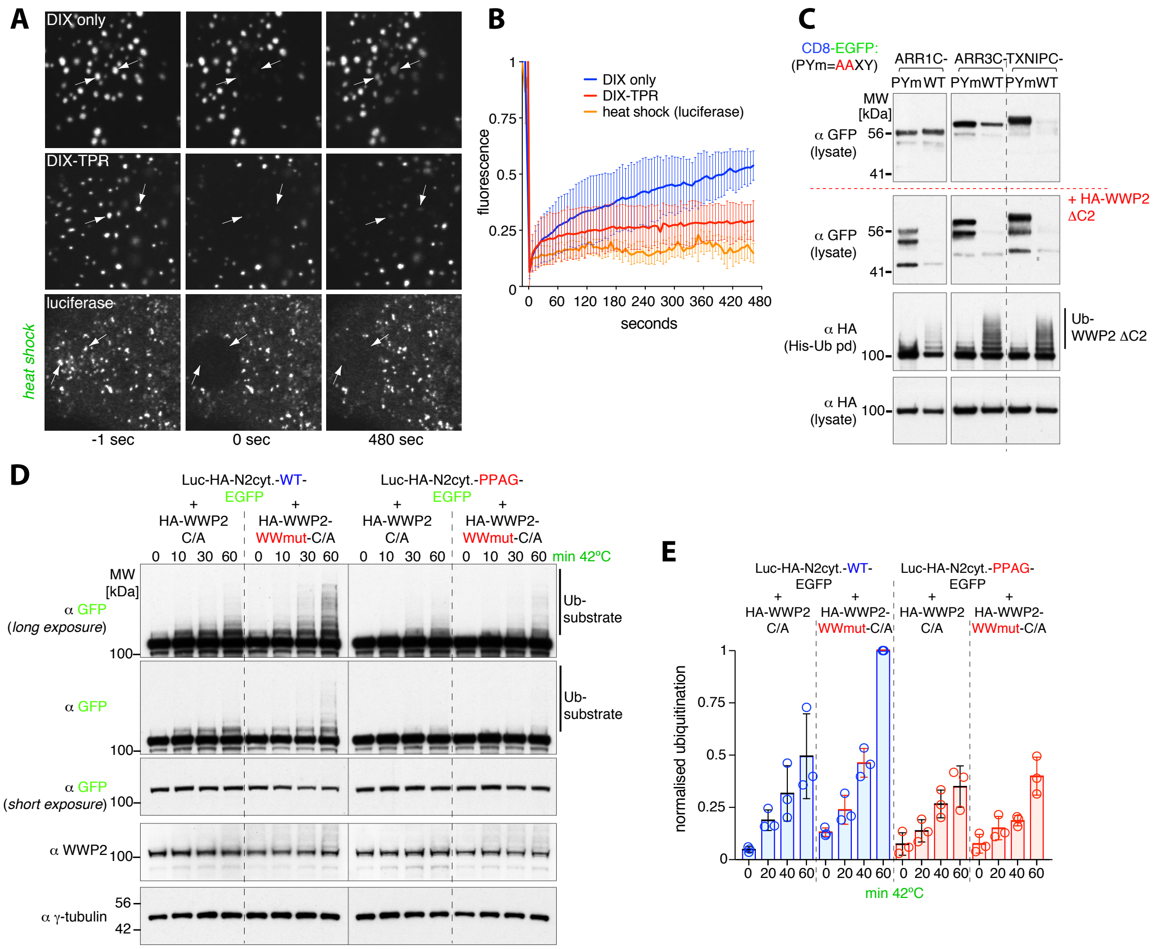

Supplementary Figure S1

(A) Representative examples of FRAP experiments (as shown in B) displaying live cell single confocal sections of HeLa cells immediately before (-1), directly after (0) or 480 seconds after photobleaching. (B) Quantitation of FRAP experiments. Each curve represents one bright dot in the bleached region. FRAP experiments were done on HeLa cells transfected with the cytoplasmic domain of Ndfip2 tagged with EGFP that was either N-terminally fused to DIX, DIX-TPR or luciferase. To induce aggregation the luciferase-transfected cells were heat shocked for 15 minutes at 43°C. (C) Western blots of His pull-downs from HEK293T cells co-transfected with His-Ub, membrane-tethered CD8-alpha arrestin constructs and without (top panel) or with HA-tagged WWP2DC2 and as indicated. This set uses a different PY mutant (AAXY) as compared to Fig.3D (PPXG) but gives the same result. (D) Western blots showing ubiquitination of the indicated luciferase constructs (with or without PY motifs) in heat shocked cells, with co-expression of a dominant negative form of WWP2 that lacks the active site cysteine (C/A) or a control in which the WW domains are also mutated (WWmut). The dominant negative reduces ubiquitination of the PY-containing construct by endogenous enzymes. Three different exposures of the anti-GFP blot are shown for clarity. (E) quantitation of experiments as shown in (d), showing actual values, mean and standard deviation for each sample.
